# Implementing Instagram as educational tool for teaching hematology and medical oncology – a cross-sectional study

**DOI:** 10.1186/s12909-026-09350-0

**Published:** 2026-05-05

**Authors:** Emmi Altmann, Jutta Hübner, Christoph Bauer-Büntzel, Judith Büntzel

**Affiliations:** 1 Department of Internal Medicine II, Hematology and Oncology, University Medical Center Jena, Jena, Germany; 2 Department of Nephrology and Hypertension, Center for Internal Medicine and Medical Clinic III, Klinikum Fulda, Fulda, Germany; 3 Department of Hematology and Medical Oncology, University Medical Center Goettingen, Goettingen, Germany

**Keywords:** Interactive learning, Instagram, Social media, Medical education

## Abstract

**Background:**

Social media platforms are widely used by medical students not only for personal communication but increasingly for educational purposes. Despite this trend, evidence on structured integration of social media into medical curricula remains limited. This study explores the use of Instagram as a supplementary asynchronous teaching tool in hematology and medical oncology.

**Methods:**

An Instagram account was created to complement the hematology and oncology teaching module. Educational content was disseminated through posts and interactive, case-based stories. Posts were categorized into five themes: ‘knowledge’, ‘media’, ‘work-life balance’, ‘patient care’, and ‘other’. Engagement rate served as a proxy for student interest. Interactive features such as polls, quizzes, and multiple-choice questions were employed to promote participation. Student interaction was quantified by story views relative to total followers. A survey was conducted at the end of the module to evaluate student perceptions.

**Results:**

Posts related to ‘work-life balance’ achieved the highest engagement, while other categories showed no significant difference in interaction. Case-based Instagram Stories were viewed by an average of 48.9% (± 7.3%) of followers, indicating consistent student engagement. Case length did not affect participation. Among interaction formats, multiple-choice questions were preferred. In the final evaluation, 98.4% (60/62) of respondents rated the case-based learning approach via Instagram Stories as good or very good, and 93.2% (55/59) rated the “ilearnonco” Instagram account as a good or very good educational tool.

**Conclusion:**

Instagram can be effectively integrated into a medical curriculum to support asynchronous, case-based learning in hematology and oncology. The high acceptance and engagement rates highlight the potential of social media as a complementary educational resource in medical education.

**Supplementary Information:**

The online version contains supplementary material available at 10.1186/s12909-026-09350-0.

## Introduction

The COVID-19 pandemic accelerated digitalization efforts in medical education, leading to the development and enhancement of novel teaching formats, including the use of Instagram. Recent analyses have demonstrated that Instagram can serve various functions such as patient education, support for patient groups, improving accessibility, and educating peers or medical students. Most current medical students already use social media and smartphones as tools for learning [1].

Instagram has proven to be a particularly effective platform for online teaching, offering an accessible means of staying connected with students. The app’s visual nature facilitates the explanation of medical content through images and diagnostic findings. It has already been implemented in teaching subjects such as pathology, plastic surgery, radiology, and hematology [2–5].

Although social media is predominantly used for self-promotion, particularly for recruiting new residents [6–10], relatively few studies have examined its potential as teaching tool within medical education [11–15]. Nonetheless, Instagram offers multiple advantages for asynchronous learning beyond its current primary applications. One such feature is Instagram Stories, which allows users to share images and videos that are visible for 24 h. Stories can incorporate interactive question formats, polls, and links to external content such as journal articles [16]. Clinical images, concise topic summaries, and formative questions can easily be combined, making Stories a promising tool for case-based teaching.

Despite the growing interest in digital tools, data on how to systematically integrate Instagram into medical education remain scarce. In this study, we demonstrate that case-based teaching via Instagram is not only feasible but also well received by medical students. Additionally, we examined which interactive question formats are most preferred and explored students’ reflections on the strengths and limitations of using Instagram as a supplementary teaching tool. The aim of this study was to evaluate (1) if certain content posted on Instagram triggers higher engagement of the participants, (2) to investigate whether case-based teaching using social media is feasible and (3) to assess the acceptance of medical students for Instagram as asynchronous teaching tool.

## Methods

### Account set up and design

A “follower” was defined as a medical student who subscribed to the Instagram account ilearnonco. “Views” referred to the number of followers who actively viewed an Instagram Story. The account was established as previously described [11] to supplement the hematology and oncology teaching module at the University Medical Center Göttingen. The innovative teaching platform offered fourth-year German medical students an optional online learning resource during their hematology and oncology clerkship. Recruitment of participants was conducted via institutional email lists, while strict confidentiality requirements precluded verification of individual Instagram identities. In accordance with ethical approval, only students’ metadata were analyzed. In the summer semester of 2022, 171 students were enrolled in the hematology and oncology course and invited to follow, of whom 145 followed the Instagram account. During the winter semester of 2022/2023, 160 students were enrolled and invited, resulting in a total follower count of 181. To ensure privacy and foster a secure learning environment, the account was set to private, and students were admitted upon request. Content posted on the account corresponded with the daily topics covered in lectures. At least one post per day was uploaded by the teaching staff, who voluntarily managed the account. In addition, case-based stories were uploaded on weekdays. The time required to manage the account, including content creation, posting, and responding to student interactions, was approximately 60–90 min per day. Whenever medical students had questions, a faculty member provided direct feedback via Instagram’s private messaging feature and clarified any queries. Approval for retrospective data analysis was obtained from the faculty’s ethic board (date February 25, 2021; approval 19/2/21, amendment date March 1, 2023, approval 17/3/23). The content was developed to reflect the lecture curriculum and was used in accompanying posts to support student learning. Case-based learning materials were similarly created based on medical cases presented within the curriculum. The lecture curriculum covers benign and malignant hematology, solid tumor oncology, diagnostics, and anticancer therapy, including supportive care. We additionally disseminated content on work-life balance during the COVID-19 pandemic, receiving positive student feedback, and promoted wellness to raise awareness of healthy work-life balance.

### Determining engagement

Follower count was recorded from the account’s baseline settings. Views per story slide were measured 24 h after upload. Engagement rate was calculated by dividing the sum of “likes” and comments per post by the number of followers. For story-based slides, engagement was defined as the number of students who participated in interactive questions divided by the number of story views.

### Categorizing posts (Instagram feed) and case-based teaching (Instagram story)

All posts were assigned to one of five primary categories. In cases where multiple categories applied, two authors (EA, JB) reached a consensus on the most appropriate classification. The five main categories were: (1) knowledge, (2) media, (3) work-life balance, (4) patient care, and (5) other. These were further subdivided into 17 subcategories to better capture the method of knowledge delivery.

- Category 1: Knowledge – general facts (A), clinical trials (B), mnemonics (C), picture puzzles (D), reviews (E), case reports (F)
- Category 2: Media – videos (G), podcasts (H), images (I), comics (J), medical database recommendations (K), clinical findings (L)
- Category 3: Work-life balance – mental health (M), motivation (N)
- Category 4: Patient care – patient care (O)
- Category 5: Other – templates (P), evaluation (Q)

While the main categories provided a general framework, subcategories specified the format or strategy used to convey content.

### Cased-based teaching using the Instagram Story

The Instagram Story feature was used for case-based learning. Multi-slide Stories began with a clinical vignette describing the main symptoms, often supplemented by relevant clinical images. Cases were classified into six thematic areas: medical oncology, benign hematology, malignant hematology, hemostaseology, transfusion medicine, and supportive care. Students were asked diagnostic and therapeutic questions throughout the case before the final diagnosis was revealed. Three question formats were employed: multiple-choice questions, binary decision questions (e.g., yes/no), and open-ended questions. For open-ended questions, explanations were provided at the end of the case. Students who submitted responses to open questions received individualized feedback via private message.

### Instagram flash poll

To evaluate the Instagram account as a teaching tool, students were invited to participate in a flash poll conducted via the Instagram Story feature. The survey comprised 14 items designed to assess: (1) acceptance of Instagram/social media as a teaching tool, (2) effectiveness of case-based learning via Stories, and (3) post content. Each item was presented with multiple-choice response options. As each question could be answered independently, response rates varied per item. The questions are listed in Table 1. Return rates were calculated by dividing the number of responses by the number of views per individual item.

**Table 1:** Questions of the Instagram flash-poll evaluation

Question	Answer Options
How do you rate ilearnonco as a learning method?	Very suitable Suitable Partially suitable Unsuitable
I can absorb the learning content conveyed on Instagram better than in lectures.	Strongly agree Somewhat agree Somewhat disagree Strongly disagree
I remember learning content better when I use Instagram instead of a book.	Strongly agree Somewhat agree Somewhat disagree Strongly disagree
I would like Instagram to be used more frequently as a learning method.	Strongly agree Somewhat agree Somewhat disagree Strongly disagree
It is easier for me to ask questions on ilearnonco than during lectures/seminars.	Strongly agree Somewhat agree Somewhat disagree Strongly disagree
Using Instagram Stories for case studies is a suitable teaching tool for problem-based learning.	Strongly agree Somewhat agree Somewhat disagree Strongly disagree
I rate the interactive use of questions in case studies as…	Very good Good Less good Poor
The difficulty level of the questions in the case studies was…	Too easy Appropriate Difficult Too difficult
Through the knowledge quizzes in the Instagram Stories, I was able to recognize my strengths and weaknesses in certain subject areas.	Strongly agree Somewhat agree Somewhat disagree Strongly disagree
Which type of question did you like best?	Multiple Choice Poll (Yes/No) Open-ended questions Only the content (clinical findings, etc.)
Which learning posts did you like best?	Studies Memory aids Clinical findings Comics/Giant Microbes
My interest in clinical studies was sparked by the study posts.	Strongly agree Somewhat agree Somewhat disagree Strongly disagree
The use of the presented memory aids made learning easier for me.	Strongly agree Somewhat agree Somewhat disagree Strongly disagree
The posts on ilearnonco increased my interest in hematology and oncology.	Strongly agree Somewhat agree Somewhat disagree Strongly disagree

### Statistical analysis

Post “likes” were recorded one week after the conclusion of the teaching module. Story views were measured 24 h after upload. Data were compiled using Microsoft Excel (Excel 2013). Statistical analyses were performed using GraphPad Prism version 9.0 (GraphPad Software, San Diego, CA, USA). As data on likes per post were not normally distributed, the Mann–Whitney U test was used to compare engagement across posting categories. For parametric data, the unpaired two-tailed Student’s t-test was applied. A p-value < 0.05 was considered statistically significant, and a p-value < 0.15 was interpreted as a trend.

Language editing of the main part of the manuscript was performed using ChatGPT (version 4.0; OpenAI, San Francisco, CA, USA) to improve clarity, grammar, and readability. The tool was used exclusively for linguistic refinement; all scientific content, interpretations, and conclusions were developed by the authors.

## Results

### Students show a preference for post containing mnemonics and information on work-life-balance

Posts uploaded fell in one of five main categories (1 – “knowledge”, 2 – “media”, 3 – “work-life-balance”, 4 – “patient care” and 5 – “others”). Overall, students did not engage more with any of the content categories, including knowledge, media, patient care or others. However, we observed a significant increase of interaction rate when comparing category 3 – work-life-balance – to categories 1, 2 or 4 (p < 0.01). We observed no difference in interaction rates concerning the five main categories when comparing interaction rates of the summer semester’s and winter semester’s cohort of students.

Main categories 1 (knowledge) and 2 (media) were further divided in 12 subcategories for sub-group analysis: A – general (medical) information, B – clinical trials, C – mnemonic, D – puzzle, E – review, F – case report, G – video, H – podcast, I – image/general pictures, J – comic, K – recommendations (data bases, online resources), L – clinical findings (e.g. computer tomography scans or X-rays). Overall, we observed a trend towards a higher preference to post belonging to the subcategory A compared to B (Mann-Whitney U- test, *p* = 0.110) or F (Mann-Whitney U- test, *p* = 0.063), and a significant preference to subcategory A compared to D (Mann-Whitney U- test, *p* = 0.035). Subcategory C was also significantly more often liked than category F (Mann-Whitney U- test, *p* = 0.017) and we observed a trend that students preferred subcategory C to subcategories D (Mann-Whitney U- test, *p* = 0.063) and L (Mann-Whitney U- test, *p* = 0.142). Subcategory F was liked more than subcategory I (Mann-Whitney U- test, *p* = 0.121). In summary, students showed the greatest interest in posts providing general medical information (A) and mnemonics (C), whereas posts on clinical trials (B) and case reports (F) attracted comparatively less engagement.

### Case-based using the Instagram Story teaching is well accepted amongst medical students

The Instagram Story feature was used for asynchronous, case-based learning. On average, Stories slides received 0.489 (± 0.073) views per follower. View rates per follower did not differ significantly between the summer and winter cohorts (Student’s t-test, *p* > 0.05). Case numbers were sufficient in the categories “medical oncology,” “malignant hematology,” and “transfusion medicine” to assess students’ category preferences; however, no significant differences were observed (Mann–Whitney U test, *p* > 0.05).

A significant drop in attention was noted when comparing the first and last slides of each Story, with 0.569 (± 0.074) views per slide and follower at the beginning versus 0.420 (± 0.052) at the end (Student’s t-test, *p* < 0.001). Nonetheless, case length (≤ 9 vs. > 9 slides) had no significant effect on participation (Student’s t-test, *p* > 0.05).

**Fig. 1:**
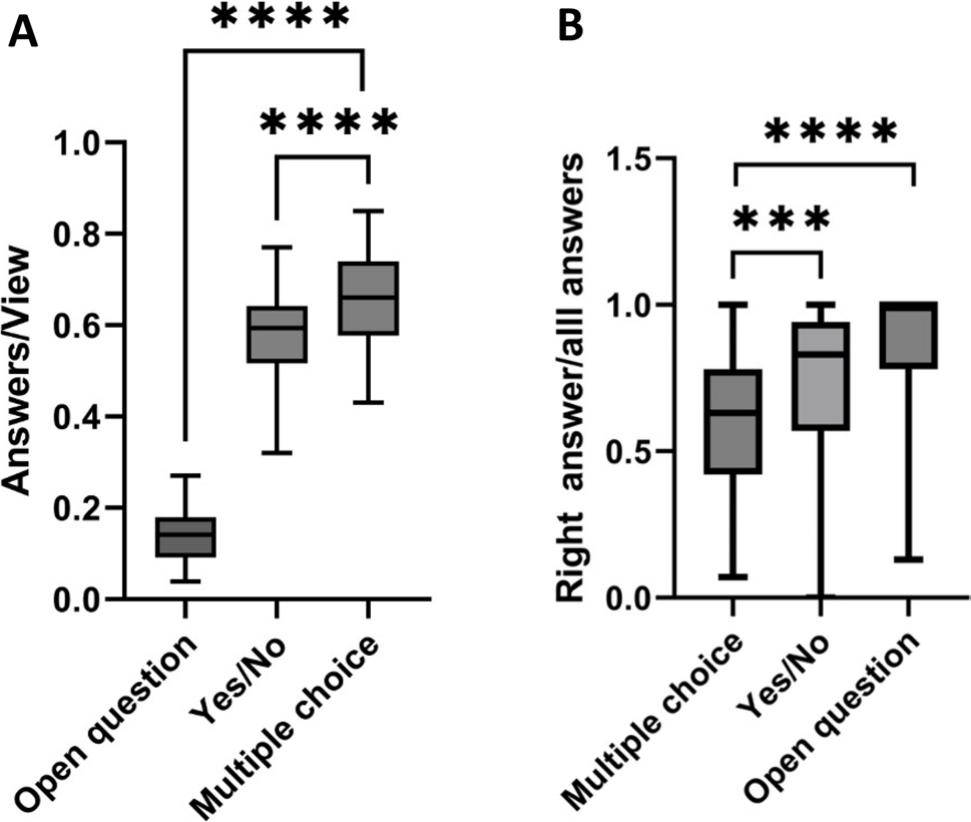
Answering behavior of students participating in online quizzes. **A** Multiple choice questions are significantly more popular than yes/no or open questions showing the highest rate of answers/views, Student’s unpaired t-test. However, **B **open questions as well as yes/no questions show a significantly higher rate of correct answers, Student’s unpaired t-test

Approximately, one out of two students viewing the questions answered (0.482 +/- 0.230 answers per view and follower). On average, one out of two students who viewed a story slide also answered the embedded question (0.482 ± 0.230 answers per view and follower). Participation rates varied by question type. Students were significantly more likely to respond to multiple-choice questions than to decision (yes/no) or open-ended questions (Student’s t-test, *p* < 0.001; Fig. 1A).

**Fig. 2:**
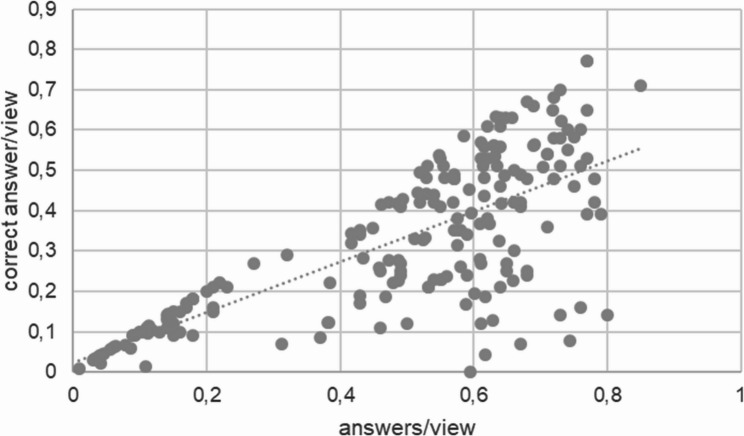
Medical students are more prone to answer a question, if they are able to answer correctly, Pearson’s *r* = 0.745, *p* < 0.00001

Regarding correctness, the highest proportion of correct answers was seen for open questions (0.885 ± 0.206 correct/total answers), followed by decision questions (0.736 ± 0.238 correct/total answers) and multiple-choice questions (0.592 ± 0.224 correct/total answers). The correctness rate for open questions was significantly higher than for both decision and multiple-choice formats (Student’s t-test, *p* < 0.001; Fig. 1B). A strong positive correlation was observed between the number of answers per view and correctness per view (Pearson’s *r* = 0.745, *p* < 0.00001), suggesting that students tended to participate when confident in their answers (Fig. 2).

### Medical students approve of Instagram as teaching tool

A total of 62 students participated in the survey, representing a subset of the 181 followers of the Instagram account. Among them, 93.2% (55/59) rated the Instagram account ilearnonco as a good teaching tool, with 42.4% (25/59) rating it as very good. Interestingly, 83.1% (49/59) reported improved knowledge retention when learning via Instagram compared to traditional lectures. A majority (83.3%, 50/60) expressed support for more frequent integration of social media into medical education.

In the second section of the survey, 98.4% (60/62) of respondents rated Instagram Stories as a good or very good format for case-based learning. Likewise, 98.4% (60/61) found the addition of interactive questions within Stories to be appropriate. Regarding preferred question types, multiple-choice questions were favored by 82.8% (48/58), compared to decision or open-ended formats.

In the third survey section, students expressed the highest approval for content related to teaching aids (e.g., mnemonics; 66.7%, 40/60), followed by clinical findings (e.g., CT scans, blood smears; 26.7%, 16/60). A majority of students (89.8%, 53/59) reported that the posts increased their interest in hematology and medical oncology.

## Discussion

Previous studies have shown that medical students have a strong interest in using social media for educational purposes. When asked, many students express a desire for the integration of social media into the medical curriculum [1, 3–5, 17]. Consistent with our findings, a survey of 1,312 medical students found that 90% used social media daily—not only for social interaction but also as a learning resource [18]. In our own survey, the majority of students indicated a clear preference for integrating Instagram into medical teaching. Similarly, a study conducted in Saudi Arabia revealed that most students believed social media enhanced their learning [19]. Although there is broad consensus in the literature that social media should be integrated into formal teaching structures, its use to actively improve learning outcomes remains limited [18]. Nonetheless, students perceive social media as a useful and engaging complement to traditional teaching formats [20, 21].

Given these findings, the question arises: why should educators not integrate this widely accepted and accessible tool into their curricula? Literature revealed that social media is already well established as a recruitment tool for medical students [6–10]. However, there remains a lack of guidance and evidence on how to systematically use social media as an effective educational platform [11, 14]. In particular, strategies for delivering relevant and pedagogically sound content via social media are still underdeveloped.

Instagram’s visual nature offers a unique advantage for disciplines reliant on visual diagnostics, such as radiology or pathology. A study conducted at Johns Hopkins Hospital demonstrated that medical illustrations and static images were among the most popular content types. However, the authors also noted difficulties in evaluating which types of posts are most effective, citing platform limitations such as the plateauing of “likes” over time [14]. While content categorization by organ system was feasible in that context, classification in hematology and oncology proved more complex.

A previous study [11] at our own institution investigated which Instagram content attracted the most student engagement. Koenig et al. reported that content related to clinical trials received the least interest, while no significant differences were found between posts focusing on teaching aids, clinical daily life, or clinical trials. Our study observed similar results: interaction rates between the categories “knowledge,” “media,” “patient care,” and “others” showed no significant differences. However, posts categorized under “work-life balance” demonstrated significantly higher engagement than posts in the “knowledge,” “media,” or “patient care” categories. These findings align with the results from Koenig et al., who observed high engagement in the “self-awareness” category, which also included content on student well-being.

As broad content categories did not allow us to draw firm conclusions about which educational materials were most effective, we implemented a subcategory structure for more detailed analysis. Consistent with earlier findings, posts related to “clinical trials” generated less engagement, while those containing teaching aids (e.g., mnemonics) and general clinical information were more well received. These results suggest that Instagram may be particularly useful for the concise repetition of clinical facts [11].

Beyond static posts, Instagram also offers interactive features such as the “Story” function, which includes quizzes, polls, and open-text questions. To evaluate its potential for interactive learning, we designed a series of clinical cases for use in the Instagram Story. Case-based learning is known to improve both knowledge acquisition and student motivation [22, 23], and our findings reflect this: approximately half of all followers regularly viewed the cases, and half of those who viewed them participated in answering the questions. Our study is among the first to demonstrate that case length does not significantly affect student engagement—cases with more than nine slides were equally well received.

Three different question formats—multiple choice, binary decision (yes/no), and open-ended—were embedded within the case narratives. Multiple-choice questions were most preferred, likely due to their prevalence in medical education assessments and their familiarity to students [24]. Overall, both multiple-choice and decision questions were well received. Open-ended questions were generally answered only when students felt confident in their response. This is consistent with broader trends in learner behavior, where students increasingly seek immediate feedback and gratification [25]. While feedback on multiple-choice and decision questions was provided instantly, open-ended responses were answered at the end of the case.

Given the exploratory nature of this study, we did not assess whether the use of the Instagram account ilearnonco led to long-term knowledge retention. However, findings from other fields suggest this may be the case. For example, a survey study in veterinary education reported that participants perceived a lasting positive impact of Instagram on both knowledge and practical skills [26]. More objective evidence comes from a randomized controlled trial by Erden et al. (2024), in which nursing students who received supplementary content via Instagram performed better in follow-up assessments compared to those who did not [27].

Nevertheless, we emphasize that such a tool cannot replace traditional clinical teaching or bedside instruction; rather, it complements these elements by providing concise, accessible reinforcement of key concepts. We also observed some variability in engagement depending on the semester, which appears to be related to workload and rotation schedules.

### Limitations

Several limitations of this study should be acknowledged. Although the total sample size of posts and cases was adequate due to the pooling of data from two semesters, subgroup analyses of both post categories and case categories were limited by small numbers. A follow-up study using a similar categorization framework with a larger dataset may yield more definitive insights.

Access to the Instagram account was restricted by setting it to private in order to create a safe and confidential learning environment. As a result, the content was only accessible to students enrolled in the hematology and oncology teaching module at our faculty. This limits the generalizability of our findings to a broader population of medical students. We also assumed a basic familiarity with social media among participants. However, it is possible that some students did not engage with the platform due to a lack of familiarity with Instagram as a teaching tool.

Another limitation of our study is that both the flash survey and the engagement metrics may reflect a self-selected subgroup of students who chose to follow and actively engage with the Instagram account. Consequently, the findings may over represent students who are already inclined to use social media as a learning tool. Moreover, interpreting “likes” as engagement is limited, as students could view and benefit from the content without actively liking posts. Finally, we cannot fully exclude the possibility that some students perceived implicit incentives—such as potential advantages for exam preparation or positive visibility to faculty— which may have influenced their engagement independently of educational interest.

## Conclusion

This study demonstrates that various features of Instagram can be effectively integrated into an academic curriculum for teaching hematology and medical oncology. Medical students showed high acceptance of the platform as a supplementary learning tool. Instagram posts proved suitable for sharing additional clinical information and teaching aids that support knowledge retention. Case-based learning via Instagram Stories was well received, with a high level of student participation. Interactive elements—particularly multiple-choice and decision-based questions—enhanced engagement and should be considered essential components when using Instagram for case-based teaching.

## Supplementary Information

Supplementary Material 1.

## Data Availability

The datasets used and/or analyzed during the current study are available from the corresponding author on reasonable request.
