# Supplementary material for: Implementing Instagram as educational tool for teaching hematology and medical oncology – a cross-sectional study
Source: BMC Med Educ. 2026 May 5;26:723. doi: 10.1186/s12909-026-09350-0 (PMC13147703; doi:10.1186/s12909-026-09350-0)
Supplement: Supplementary file 1 — Supplementary Material 1. [file 12909_2026_9350_MOESM1_ESM.docx]

Questions of the Instagram flash-poll evaluation

| Question | Answer Options |
| --- | --- |
| How do you rate ilearnonco as a learning method? | Very suitable Suitable Partially suitable Unsuitable |
| I can absorb the learning content conveyed on Instagram better than in lectures. | Strongly agree Somewhat agree Somewhat disagree Strongly disagree |
| I remember learning content better when I use Instagram instead of a book. | Strongly agree Somewhat agree Somewhat disagree Strongly disagree |
| I would like Instagram to be used more frequently as a learning method. | Strongly agree Somewhat agree Somewhat disagree Strongly disagree |
| It is easier for me to ask questions on ilearnonco than during lectures/seminars. | Strongly agree Somewhat agree Somewhat disagree Strongly disagree |
| Using Instagram Stories for case studies is a suitable teaching tool for problem-based learning. | Strongly agree Somewhat agree Somewhat disagree Strongly disagree |
| I rate the interactive use of questions in case studies as… | Very good Good Less good Poor |
| The difficulty level of the questions in the case studies was… | Too easy Appropriate Difficult Too difficult |
| Through the knowledge quizzes in the Instagram Stories, I was able to recognize my strengths and weaknesses in certain subject areas. | Strongly agree Somewhat agree Somewhat disagree Strongly disagree |
| Which type of question did you like best? | Multiple Choice Poll (Yes/No) Open-ended questions Only the content (clinical findings, etc.) |
| Which learning posts did you like best? | Studies Memory aids Clinical findings Comics/Giant Microbes |
| My interest in clinical studies was sparked by the study posts. | Strongly agree Somewhat agree Somewhat disagree Strongly disagree |
| The use of the presented memory aids made learning easier for me. | Strongly agree Somewhat agree Somewhat disagree Strongly disagree |
| The posts on ilearnonco increased my interest in hematology and oncology. | Strongly agree Somewhat agree Somewhat disagree Strongly disagree |
